# The Utility of Next-Generation Sequencing for Identifying the Genetic Basis of Dementia

**DOI:** 10.3390/ijerph18168520

**Published:** 2021-08-12

**Authors:** Aleksandra Klimkowicz-Mrowiec, Anna Dziubek, Małgorzata Sado, Marek Karpiński, Agnieszka Gorzkowska

**Affiliations:** 1 Department of Internal Medicine and Gerontology, Faculty of Medicine, Medical College, Jagiellonian University, 30-688 Krakow, Poland; mkarpinski@su.krakow.pl; 2 Haematological Diagnostics and Genetics Unit, University Hospital, 30-688 Krakow, Poland; adzi@su.krakow.pl (A.D.); msado@su.krakow.pl (M.S.); 3 Department of Neurorehabilitation, Faculty of Medical Sciences, School of Medicine, Medical University of Silesia, 40-752 Katowice, Poland; agorzkowska@sum.edu.pl

**Keywords:** Alzheimer’s disease, frontotemporal dementia, next generation sequencing, biomarkers, psychiatric disorder

## Abstract

The clinical manifestations of dementia are often rapidly matched to a specific clinical syndrome, but the underlying neuropathology is not always obvious. A genetic factor often plays an important role in early onset dementia, but there are cases in which the phenotype has a different genetic basis than is assumed. Two patients, at different times, presented to the Memory Clinic because of memory problems and difficulty in performing daily activities and work. Neither caregiver complained of marked behavioural or personality changes, except for apathy. Patients underwent standard dementia evaluation procedures including clinical symptoms, family history, neuroimaging, neuropsychological evaluation, and genetic analysis of selected genes. Based on specific clinical phenotypes and genetic analysis of selected genes, both patients were diagnosed with frontal variant of Alzheimer’s disease. The presence of a rare polymorphism in *PSEN2* in both patients allowed the discovery that they belong to the same family. This fact reinforced the belief that there is a strong genetic factor responsible for causing dementia in the family. Next-generation sequencing based on a panel of 118 genes was performed to identify other potential genetic factors that may determine the background of the disease. A mutation in the *GRN* gene was identified, and the previous diagnosis was changed to frontotemporal dementia. The described cases show how important it is to combine all diagnostic tests available in the diagnostic centre, including new generation genetic tests, in order to establish/confirm the pathological background of clinical symptoms of dementia. If there is any doubt about the final diagnosis, persistent efforts should be made to verify the cause.

## 1. Introduction

The primary challenge for the clinician caring for patients with dementia is to identify the clinical syndrome that best matches the presenting symptoms and confirm its molecular basis. Clinical symptoms are often quickly matched to a specific clinical syndrome, whereas the underlying neuropathology is not always obvious. We describe two patients from a single family who were initially diagnosed with frontal variant of Alzheimer’s disease (fvAD) based on a history and additional examinations. Genetic evaluation was focused on the selected genotypes (*ApoE*, *APP*, *PSEN1*, *PSEN2*). A variant in one allele of *PSEN2* gene was revealed, classified as benign/likely benign. Because the pedigree suggested a strong genetic basis for the symptoms and the pathogenicity of the identified mutation in *PSEN2* was uncertain, we decided to use Next-Generation Sequencing (NGS). This technology allows efficient analysis of several genes simultaneously, especially in the diagnosis of diseases in which different genes are responsible for the same clinical phenotype, such as in dementing disorders [1].

## 2. Materials and Methods

### 2.1. Case 1

A 59-years-old woman, a cook, presented to the Outpatient Clinic. The first symptoms, according to her husband, began a year earlier in the form of memory problems, the patient quickly forgot new information, did not attend appointments, and had difficulties with everyday activities such as cooking (she often left the pot on the stove burning the food). The patient had been fired from her job several months earlier because she was unable to fulfil her responsibilities. Other than apathy, no other behavioural disturbances were observed. Her family history showed that her brother, 5 years older, had been diagnosed with Alzheimer’s disease several years earlier. The patient’s mother (who died at the age of 84) had suffered from dementia for 10 years. The results of the neurological examination were normal. The subject scored 23/30 on the Mini Mental State Examination (MMSE) (she was not oriented to the month, date, lost 3 points when counting backwards and 2 when recalling words after distraction), and 10/10 on the Clock Drawing Test (CDT).

**Figure 1:**
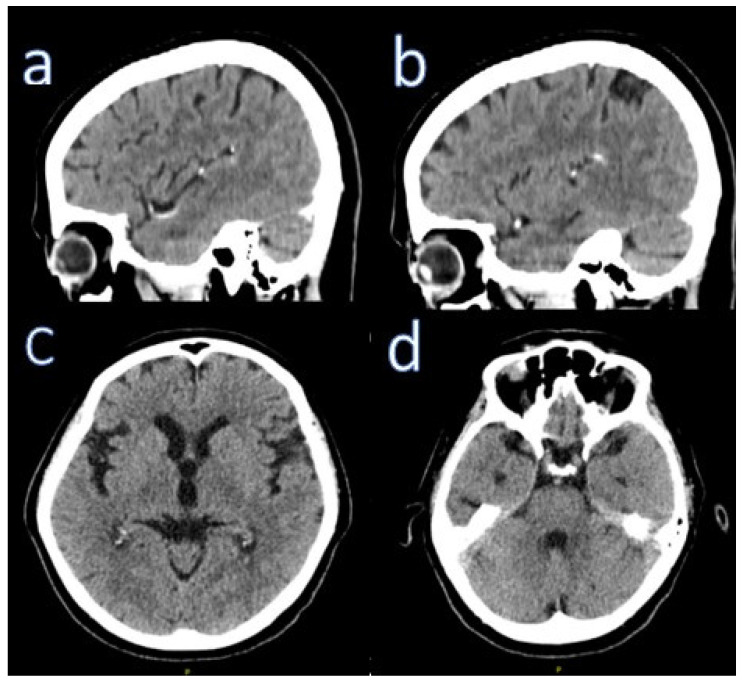
CT of Case 1 taken after first presentation in outpatients’ clinic. Sagittal (**a**,**b**) and axial (**c**,**d**) CT projections.

On follow-up examination, all laboratory results were normal. A CT scan of the brain (the patient did not consent to the MR scan due to claustrophobia) showed slight subcortical and cortical atrophy in the frontal, temporal, and occipital lobes (Figure 1).

The neuropsychological profile of cognitive impairment showed episodic memory impairment and executive dysfunction. Molecular genetic analyses of the *PSEN1*, *PSEN2*, *APP*, *APOE* genes were performed by Sanger sequencing method. The patient had *APOE3/3* genotype. A variant c.185G>A in one allele of *PSEN2* gene was found. This variant was classified as benign/likely benign in ClinVar NCBI. In the Human Gene Mutation Database (HGMD), the patient with sporadic AD carrying this variant was registered [CM981662]. The pathogenic role of the *PSEN2* Arg62His mutation has not been demonstrated in a family with frontotemporal dementia (FTD), where the variant was found in an asymptomatic mother and her symptomatic daughter, and the authors argued that other factors, including *APOE* genotype, may influence the onset of the disease in carriers of the variant [2]. The variant has been reported as a common variant in African populations [3] and, after in vivo functional analysis, as a rare polymorphism with no effect on neurodegeneration [4]. On the other hand, this variant has also been shown to affect the stability of the protein, making it more susceptible to degeneration [5].

Based on the specific clinical phenotype (predominant executive function impairment, in addition to impairment of episodic memory in cognitive tests and the presence of apathy) and the results of genetic testing, the patient was diagnosed with frontal variant Alzheimer’s disease [6].

### 2.2. Case 2

Fourteen months later, a 55-year-old man, a blue-collar worker, presented to the Outpatient Clinic because of memory impairment. The patient’s wife had noticed the first symptoms 4 years before the visit: memory problems, disorientation in space, and difficulty handling money. The patient was still independent at home for simple activities of daily living but required supervision for more complex tasks because he immediately forgot what he was doing. His wife complained that the patient was very apathetic. She reported no other behavioural disturbances. The patient’s mother, who was 83 years old at the time, had had memory problems for 5 years and required constant care from others.

**Figure 2:**
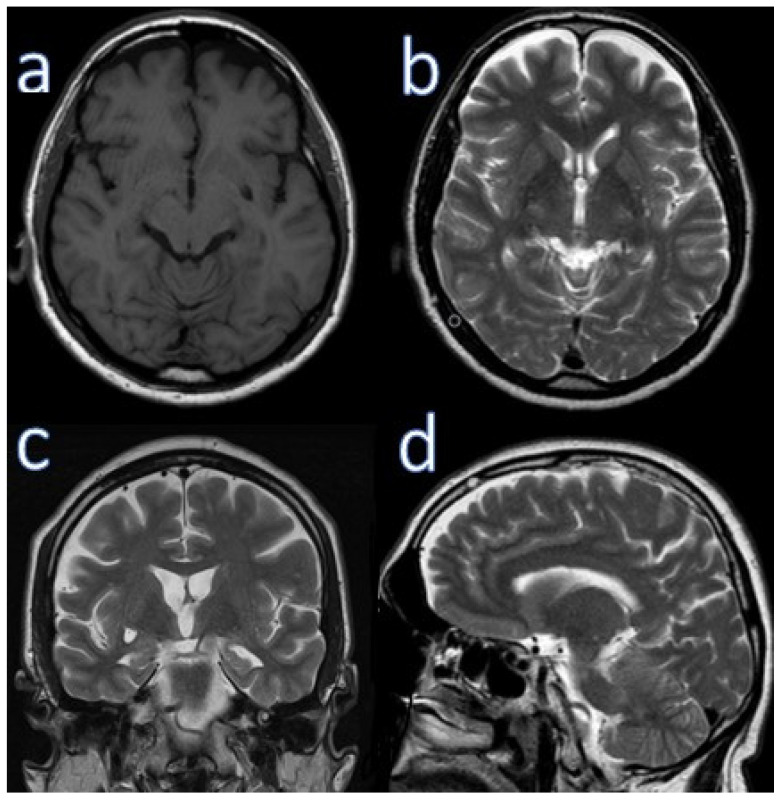
Brain MRI of case 2. (**a**) Axial T1 weighted, (**b**) axial T2 weighted (upper row). (**c**) Coronal T2 weighted, (**d**) sagittal T2 weighted (lower row).

Neurological examination results were normal. He scored 20/30 on MMSE (he did not orient to time, lost 3 points when counting backwards and 3 points when recalling words after distraction), and 10/10 on the CDT. A head MR scan performed 17 months before presentation showed frontoparietal cortical atrophy, small disseminated vascular lesions up to 6 mm in the frontal and occipital lobes, and an enlarged Virchow-Robin space in the left globus pallidus (5 × 10 mm) (Figure 2).

At follow-up, all laboratory findings were normal. Neuropsychological evaluation revealed episodic memory impairment and executive dysfunction. Routine molecular genetic analysis of the *PSEN1*, *PSEN2*, *APP*, *APOE* genes were performed by Sanger sequencing. A variant in one allele of *PSEN2* gene c.185 G > A was revealed. The patient had *APOE3/3* genotype. Based on the specific clinical phenotype (predominant executive function impairment, in addition to impairment of episodic memory in cognitive tests and the presence of apathy) and the results of genetic testing, the patient was diagnosed with frontal variant Alzheimer’s disease [6].

The polymorphism in the PSEN2 gene found in both cases is so rare that we started investigating whether the two patients were related. They turned out to be second line cousins.

At the same time, the outpatient clinic gained access to NGS. Due to the strong suspicion of the genetic basis for dementia in the family and weak data on the pathogenicity of detected variant in *PSEN2*, the patient’s DNA was sent for further testing. A panel of 118 genes associated with neurogenetic disorders (based on HPO, OMIM, Genereviews, Orphanet) was used, for which a library was prepared using the KAPA Hyper Plus kit (Roche), sequencing was performed on MiSeq platform using the MiSEq reagent Kit v3 (150 cycles) (illumina), and the results were analyzed using BaseSpace Variant Interpreter and Integrative Genomics Viewer (IGV) (average read depth was 119.2×; 96.2% sequences was covered more than 30×).

The work was conducted in accordance with the Helsinki Declaration. All patients gave their written consent for genetic testing. Written informed consent was obtained from the patient for publication of this case report and any accompanying images.

## 3. Results

Both patients had a mutation in one allele of *GRN* gene c.138 + 1 G > A. This mutation has already been reported as pathogenic in LOVD, ClinVar NCBI (RCV001049316), dbSNP NCBI (rs63749844) and HGMD-PUBLIC (CS064416) genetic variant databases, but was not found in the Genome Aggregation Database (which is based on both disease-specific and general population genetic studies). It has also been described in two cohort genetic studies [7,8]. In one study, patient’s frontal cortices were available as a source of mRNA to study the effect of c.138 + 1 G > A mutation. Transcript analysis by RT–PCR in a patient carrying the c.138 + 1 G > A mutation revealed the presence of an aberrant product corresponding to exon 1 skipping (268 bp) in addition to the wild-type transcript (413 bp). The exclusion of exon 1 containing the start methionine codon from the progranulin mRNA is expected to block progranulin protein from being generated, creating a functional null allele [8].

**Figure 3:**
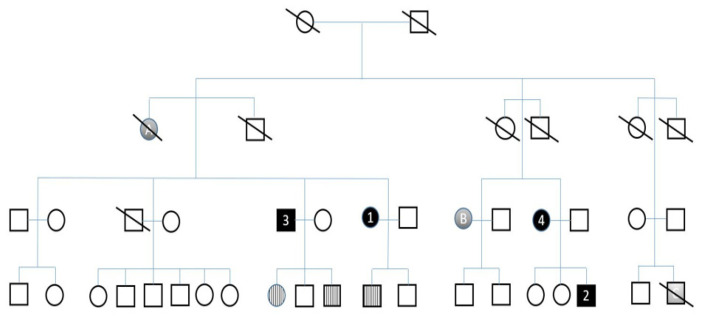
Pedigree of a family with frontotemporal dementia.

Blood samples for DNA testing were collected from nine other available family members, who gave written informed consent. The same mutation was identified in two individuals with dementia (brother of case 1, mother of case 2), as well as in asymptomatic children of individuals with dementia. Figure 3 shows the members of the described family.

Cross-hatched symbols represents individuals who are no longer living. Black circles (females) and squares (males) represent individuals with dementia (1,2,3,4). Symbols with colour gradients represent patients with a positive history of dementia without genetic testing (A,B,C). Symptomless individuals who are mutation carriers are in the pattern.

## 4. Discussion

Proper clinical differentiation between fvAD and bvFTD has implications for prognosis, treatment, and caregiver burden. Unfortunately, the clinical presentation in the course of bvFTD and fvAD can be very similar. FvAD is characterized by episodic memory impairment accompanied by behavioural and/or executive function deficits [6]. The clinical course of FvAD is less well defined, coming mainly from case reports.

Our patients’ history and additional testing first suggested fvAD (dysexecutive type), but extended genetic testing eventually revealed another cause of dementia. After receiving the NGS results, we asked ourselves whether it was possible to make a correct clinical diagnosis based on the data available so far.

Both patients did not meet the diagnostic criteria for bvFTD, which include a wide range of neuropsychiatric behaviours (disinhibited behavior, profound apathy, loss of empathy, perseverative/compulsive behaviour, hyperorality or dietary changes) [9]. In both patients, the leading clinical feature was cognitive decline (episodic memory deficit, impaired learning of new information, and executive deficits). Atypical clinical presentation is more common in early-onset AD (EOAD). Apathy, which was the only neuropsychiatric disorder, is widespread in AD patients. Furthermore, in early dementia, memory impairment and executive deficits may be more common than behavioural changes.

Symptoms of dementia appeared in both patients before the age of 60 years, but in the mother of Case 2, at the age of 80 years, with episodic memory deficit initially present. Unfortunately, the age of onset of symptoms does not differentiate FTD from AD. Of all AD patients, approximately 10% are diagnosed with EOAD, with symptoms first appearing between the age of 30 to 65 years. EOAD is an almost entirely genetically determined disease, mutation determines disease onset in most families, but rare, high-penetrance mutations in *APP*, *PSEN1*, and *PSEN2* genes explain only a small percentage of families with EOAD, leaving a large group of autosomal dominant pedigrees genetically unexplained [10]. Age of onset in genetic forms of FTD is also highly variable, but unlike EOAD, with advanced within-family variability [11].

It was been shown that frontal-parietal cortical areas are involved in neuroimaging studies in AD patients more often than in patients with typical AD, but less often than in patients with bvFTD [4]. In our patients, we observed small fronto-parietal atrophy with widening of Sylvian fissure. Temporal horns dilatations and white matter hyperintensity (T2-weighted MR) were present on MR of Case 2. White matter abnormalities and frontoparietal grey matter atrophy have been observed in FTD patients, especially in *GRN* mutation carriers.

Both patients had two alleles of *APOE3*. Previous studies suggest that having *APOE4* causes phenotypic differences between *APOE4*+ vs. *APOE4*− AD patients. *APOE4*+ individuals have relatively greater tau accumulation and brain atrophy in the medial temporal lobe, resulting in greater memory impairment than *APOE4*− AD patients. On the contrary, *APOE4*− patients have relatively greater tau accumulation and brain atrophy in the frontoparietal lobes, resulting in greater impairment of executive function, visuospatial ability, and language than *APOE4*+ AD patients [12]. These observations supported our initial diagnose of AD.

The clinical data pointing to a genetic background of dementia were very strong, so we applied NGS testing to see if no other genetic factors could better explain the dementia underlying our cases that identified mutation in *PSEN2*. NGS is a powerful method to study the genetic basis of clinical symptoms, although it still has some limitations, mainly related to the large insertion/deletion detection. The identification of novel variants by NGS and use in silico analyses is useful to predict the impact that each variant may have on the transcriptome, but the results of such analyses should be treated with caution and their ultimate role determined by additional clinical data and segregation analysis of family pedigree with individuals in multiple generations who have phenotype and genotype information [13]. Confirmation of the genetic test is also important and current best practice in many laboratories is to confirm NGS with Sanger sequencing.

The results did not confirm the earlier diagnoses and donepezil was discontinued.

During the next four years of follow-up, behavioural disturbances remained minor. Case 1, in addition to apathy, had a tendency to impulsive behaviour (excessive shopping). She reached late-stage of dementia and requires ongoing care. Case 2 is still only apathetic, can be safely left at home, but requires supervision in all daily activities.

A limitation of our study is that the CSF biomarkers (Aβ1-42, t-tau, p-tau) and amyloid β imaging were not evaluated. These diagnostic procedures are difficult to access and are not reimbursed by our health care system.

## 5. Conclusions

The history of our patients demonstrates that, in detecting the causes of dementia, especially atypical dementia, one should strive to confirm the clinical manifestations of the disease with reliable biomarkers and that NGS can be very useful when other highly specific methods are not available.

## Data Availability

Not applicable.
